# HLA‐A*24:02 increase the risk of allopurinol‐induced drug reaction with eosinophilia and systemic symptoms in HLA‐B*58:01 carriers in a Korean population; a multicenter cross‐sectional case‐control study

**DOI:** 10.1002/clt2.12193

**Published:** 2022-09-15

**Authors:** Mi‐Yeong Kim, James Yun, Dong‐Yoon Kang, Tae Hee Kim, Min‐Kyung Oh, Sunggun Lee, Min‐Gyu Kang, Young‐Hee Nam, Jeong‐Hee Choi, Min‐Suk Yang, Seung Seok Han, Hajeong Lee, Hyun‐Jai Cho, Jaeseok Yang, Kook‐Hwan Oh, Yon Su Kim, Jae Woo Jung, Kye Hwa Lee, Hye‐Ryun Kang

**Affiliations:** ^1^ Department of Internal Medicine Busan Paik Hospital Inje University College of Medicine Busan Korea; ^2^ Department of Immunology and Rheumatology Nepean Hospital The University of Sydney Sydney Australia; ^3^ Drug Safety Monitoring Center Seoul National University Hospital Seoul Korea; ^4^ Department of Pharmacology Inje University College of Medicine Busan Korea; ^5^ Department of Internal Medicine Haeundae Paik Hospital Inje University College of Medicine Busan Korea; ^6^ Department of Internal Medicine Chungbuk National University Hospital Cheongju Korea; ^7^ Department of Internal Medicine Dong‐A University College of Medicine Busan Korea; ^8^ Department of Internal Medicine Hallym University Dongtan Sacred Heart Hospital Hallym University College of Medicine Hwaseong Korea; ^9^ Department of Internal Medicine SMG‐SNU Boramae Medical Center Seoul Korea; ^10^ Department of Internal Medicine Seoul National University College of Medicine Seoul Korea; ^11^ Department of Internal Medicine Yonsei University College of Medicine Severance Hospital Seoul Korea; ^12^ Department of Internal Medicine Chung‐Ang University College of Medicine Seoul Korea; ^13^ Department of Information Medicine Asan Medical Center Seoul Korea; ^14^ Institute of Allergy and Clinical Immunology Seoul National University Medical Research Center Seoul National University College of Medicine Seoul Korea

**Keywords:** allopurinol, drug hypersensitivity syndrome, histocompatibility antigens class I, HLA‐A24 antigen, Koreans

## Abstract

**Background:**

HLA‐B*58:01 is a well‐known risk factor for allopurinol‐induced severe cutaneous adverse reactions (SCARs). However, only a minority of HLA‐B*58:01 carriers suffer SCARs after taking allopurinol. The aim of this study was to investigate subsidiary genetic markers that could identify those at further increased risk of developing allopurinol‐induced drug reaction with eosinophilia and systemic symptoms (DRESS) in subjects with HLA‐B*58:01.

**Methods:**

Subjects with B*58:01 were enrolled (21 allopurinol‐induced DRESS and 52 allopurinol‐tolerant control). HLA‐A, ‐B, ‐C and ‐DRB1 alleles were compared. Comparison of risk between HLAs and allopurinol‐induced SCAR in separate populations was performed to support the results. Kruskal‐Wallis test, Pearson's chi‐square test, Fisher's exact test and binary logistic regression were used to analyze the risk of SCAR development.

**Results:**

Frequencies of A*24:02 (71.4 vs. 17.3%, *p* < 0.001, odds ratio [OR] = 12.0; 95% confidence interval [CI], 3.6–39.2) were significantly higher in B*58:01 (+) DRESS than B*58:01 (+) tolerant controls. In addition, DRB1*13:02 further increased the risk of DRESS. The phenotype frequency of A*24:02/DRB1*13:02 was significantly higher in the B*58:01 (+) DRESS group than in the B*58:01 (+) tolerant controls (52.4% vs. 5.8%, *p* < 0.001, OR, 66.0; 95% CI, 6.1–716.2). In 2782 allopurinol user cohort, the overall prevalence of DRESS was 0.22%, which increased to 1.62% and 2.86% in the presence of B*58:01 and B*58:01/A*24:02, respectively.

**Conclusion:**

The additional secondary screening with A*24:02 and DRB1*13:02 alleles may identify those at further increased risk of allopurinol‐induced DRESS in B*58:01 carriers.

## 1 BACKGROUND

Allopurinol, a xanthine oxidase inhibitor, is recommended as the first‐line urate‐lowering agent in patients with normal renal function according to the 2016 updated evidence‐based recommendation.^1^, ^2^, ^3^, ^4^ According to a recent study, allopurinol might be safer than febuxostat, especially in those with cardiovascular disease.^5^

Allopurinol has been regarded as a relatively safe drug since its launch in the 1960s, but it has the potential to induce severe cutaneous adverse reactions (SCARs), such as a drug reaction with eosinophilia and systemic symptoms (DRESS) and Stevens‐Johnson syndrome (SJS)/toxic epidermal necrolysis (TEN).^6^, ^7^ The known risk factors of allopurinol‐induced SCARs are HLA (human leukocyte antigen)‐B*58:01, Asian ethnicity, old age, female, a higher initiating dose of allopurinol, and comorbidities such as cardiovascular disease and renal impairment.^8^ Out of these, B*58:01 is the strongest risk factor.

Because the distribution of HLA alleles is different in ethnic groups,^9^ the risk of developing SCARs is affected by ethnicity.^10^ About 12% of the general population in Korea carries B*58:01, and this likely explains why allopurinol has been the most frequently reported culprit drug of SCARs.^11^, ^12^, ^13^ The usefulness of B*58:01 screening in preventing allopurinol‐induced SCARs was successfully proven in Korean patients with renal impairment in a prospective study.^14^ However, allopurinol‐induced SCARs developed only in 18% of B*58:01 positive high‐risk subjects with chronic renal insufficiency.^13^ The majority of B*58:01 carriers can safely take allopurinol without hypersensitivity reactions. A recent retrospective cohort study with 11 cases of allopurinol‐induced SCARs in Korea reported that the secondary serotype screening may help to increase the accuracy of the prediction of allopurinol‐induced SCARs.^4^

The aim of the study was to improve the screening method to predict the risk of allopurinol‐induced SCARs among subjects with B*58:01.

## 2 MATHODS

### 2.1 Study population

#### 2.1.1 Analyzing HLA‐A, ‐B, ‐C, ‐DRB1 in cases and controls with B*58:01

We identified patients who were diagnosed with ‘allopurinol‐induced SCARs (DRESS or SJS/TEN)’ from the Korean SCAR registry.^15^ A drug (allopurinol) causality evaluation was performed by allergists using the WHO (World Health Organization)‐Uppsala Monitoring Center criteria.^16^ The “certain” and “probable” cases were considered as acceptable for inclusion. ‘Allopurinol tolerant controls’ were identified subjects who had taken allopurinol for more than 90 days without any hypersensitivity symptoms. All subjects were finally enrolled when they confirmed by medical records (pre‐tested HLA information) or new HLA analyses of blood sample as B*58:01 carriers.

We collected clinical information such as age, gender, body mass index (BMI), baseline estimated glomerular fractional rate (eGFR), dose of allopurinol, and comorbidities, such as cardiovascular diseases. eGFR was calculated by the Chronic Kidney Disease Epidemiology Collaboration (CKD‐EPI) creatinine equation. Chronic renal impairment (CRI) was defined by a baseline eGFR <60 ml/min/1.73 m^2^. Because most SCAR patients had been transferred to the allergists from outside clinics without previous baseline laboratory results, information regarding eGFR before taking allopurinol was not available. Therefore, the maximum eGFR value was used as the baseline eGFR after the complete resolution of the SCAR. In the SCAR group, the values for age and BMI at the time of diagnosis were used. In the control group, the values for age, BMI, and baseline eGFR at the time of starting allopurinol were selected.

#### 2.1.2 Validation in different populations

Additional comparisons in separate populations were designed to support the results. Since the information of other HLA distribution among B*58:01 carriers in Korean general population were not available, the HLA information of 11998 HLA‐tested subjects included in the data warehouse of the Seoul National University Hospital were used to define B*58:01 (+) general population control.^17^ The first validation set was HLA‐tested allopurinol user cohort among 11998 HLA‐tested subjects. The history of allopurinol exposure and hypersensitivity were collected from the electronic medical records and adverse drug reaction records. The second population was the data of a previous multicenter case‐control study performed in Koreans, which included 25 cases of allopurinol‐induced SCARs (20 cases of DRESS and five cases of SJS/TEN).^12^ Among them, the B*58:01 (+) allopurinol‐induced DRESS were compared with the B*58:01 (+) general population control. All subjects were validated in order not to be overlapped between validation sets.

### 2.2 DNA isolation and HLA typing

Genomic DNA was extracted from blood samples using the Mini 80 nucleic acid isolation instrument with the QuickGene DNA whole blood kit S (FUJIFILM Co., Tokyo, Japan) and QIAamp® DNA Blood Mini Kit (QIAGEN, Hilden, Germany). Genotyping of HLA‐A, ‐B, ‐C, and ‐DRB1 was done with the AVITATM HLA‐SBT kits (Biowithus Inc., Seoul, Korea), as described in the manufacturer's instructions. Exons 2–4 of Class I and exon 2 of HLA‐DRB1 were amplified using a locus specific primer set in the kit. PCR products were sequenced directly in both the forward and reverse directions of each exon on an ABI3730 Genetic Analyzer (Applied Biosystems, Foster City, CA), and the sequenced results were analyzed with the BIOWITHUS SBT analyzer software ver. 2.7.5 (Biowithus Inc.).

### 2.3 Statistical analysis

Statistical analyses were performed with SPSS version 26.0 (IBM Co., NY, USA). Independent Kruskal‐Wallis test was used to analyze continuous variables. Pearson's chi‐square test and Fisher's exact test were used to analyze categorical variables. The false discovery rate (FDR)‐adjusted *Q*‐values were calculated in comparing multiple HLA phenotypes. Binary logistic regression was done to adjust clinical factors or presence of certain HLAs between groups. Results were considered significant when the *P* or *Q*‐value was <0.05.

## 3 RESULTS

### 3.1 Demographics and clinical features of the study subjects with B*58:01

We enrolled 27 B*58:01 carriers diagnosed with allopurinol‐induced SCARs (21 DRESS patients and 6 SJS/TEN patients) and 52 allopurinol‐tolerant B*58:01 carriers (Table S1).

Demographics and clinical features are described in Table 1. The median age (years) was older in the DRESS (57.5, range 13–89, *p* = 0.046) and the SJS/TEN (75.5, range 27–86, *p* = 0.021) groups than the tolerant controls (49.5, range 8–72). When compared with the tolerant controls, the proportion of males was lower in the DRESS group (71.2% vs. 35.0%, *p* = 0.003), while it was similar in the SJS/TEN group (71.2% vs. 50.0%, *p* = 0.075). There was no significant difference between the BMI and the presence of cardiovascular diseases such as hypertension, heart failure or arrhythmia. The median baseline eGFR (ml/min/1.73 m^2^) in the tolerant controls (29.0, range 2.9–78.0) was lower compared with the DRESS (38.8, range 3.2–132.6, *p* = 0.026) or SJS/TEN group (56.6, range 31.7–120.6, *p* = 0.005). The median maintenance daily dose of allopurinol was relatively lower in the tolerant controls compared to the DRESS or SJS/TEN.

### 3.2 Genetic heterogeneity between DRESS and SJS/TEN groups with HLA‐B*58:01

When the DRESS and SJS/TEN groups with B*58:01 were compared, there were no significant differences in the demographic characteristics. To assess the difference in the genetic predisposition of HLA alleles according to the SCAR phenotypes, we compared the phenotype frequency of the HLA alleles presenting at least 30% and 2‐folds difference between the DRESS and SJS/TEN groups: A*24:02, DRB1*13:02, A*02:06, C*04:01 and DRB1*04:06 met the criteria. The phenotype frequency of A*24:02 was higher in the DRESS (71.4%) than in the SJS/TEN (16.7%) group (Odds ratio [OR], 12.5, *p* = 0.027, 95% confidence interval [CI] 1.2–130.6). Phenotype frequencies of the A*02:06, C*04:01 and DRB1*04:06 were higher in the SJS/TEN than in the DRESS group (A*02:06, *p* = 0.043, OR 0.7, 95% CI, 0.4–1.2; C*04:01, *p* = 0.025, OR 0.1, 95% CI, 0.0–0.7; DRB1*04:06, *p* = 0.043, OR 0.7, 95% CI, 0.4–1.2). All FDR‐adjusted *Q*‐values of comparison by A*24:02, A*02:06, C*04:01 and DRB1*04:06 were 0.043.

### 3.3 Comparison of the HLA phenotype frequency of DRESS to allopurinol‐tolerant controls and B*58:01 (+) general population controls

Considering the possibility of genetic differences between DRESS and SJS/TEN, the comparison with tolerant controls were performed by each group. When comparing all HLA phenotype frequencies between DRESS and the tolerant controls, A*24:02 showed a significant difference (Table S2). After Bonferroni correction, A*24:02 still showed a significant difference (*p* < 0.05). The phenotype frequency of A*24:02 was significantly higher in the DRESS group than in the tolerant controls (71.4% vs. 17.3%, *p* < 0.001, OR 12.0, 95% CI, 3.6–39.2) (Table 2). In comparison with 1321 B*58:01 (+) general population controls, the phenotype frequency of A*24:02 was still significantly higher in allopurinol‐induced DRESS with HLA‐B*58:01 (71.4% vs. 17.2%, *p* < 0.001, OR 12.0, 95% CI, 4.6–31.3). We further analyzed to see whether any combination of HLA phenotypes that were predominant in DRESS compared to SJS/TEN could increase the predictability of DRESS development. The presence of DRB1*13:02 further increased the risk of DRESS development in those carrying both B*58:01 and A*24:02. The phenotype frequency of B*58:01/A*24:02/DRB1*13:02 was significantly higher in the DRESS group than in the tolerant controls (52.4% vs. 5.8%, *p* < 0.001, OR 66.0, 95% CI, 6.1–716.2).

### 3.4 Validation of HLA phenotype frequency in different data sets

Among 11,998 HLA‐tested subjects from the data warehouse of the Seoul National University Hospital, 1321 (11%) subjects with B*58:01 was defined as B*58:01 (+) general population control, and 2782 subjects were defined as allopurinol user cohort.

**FIGURE 1:**
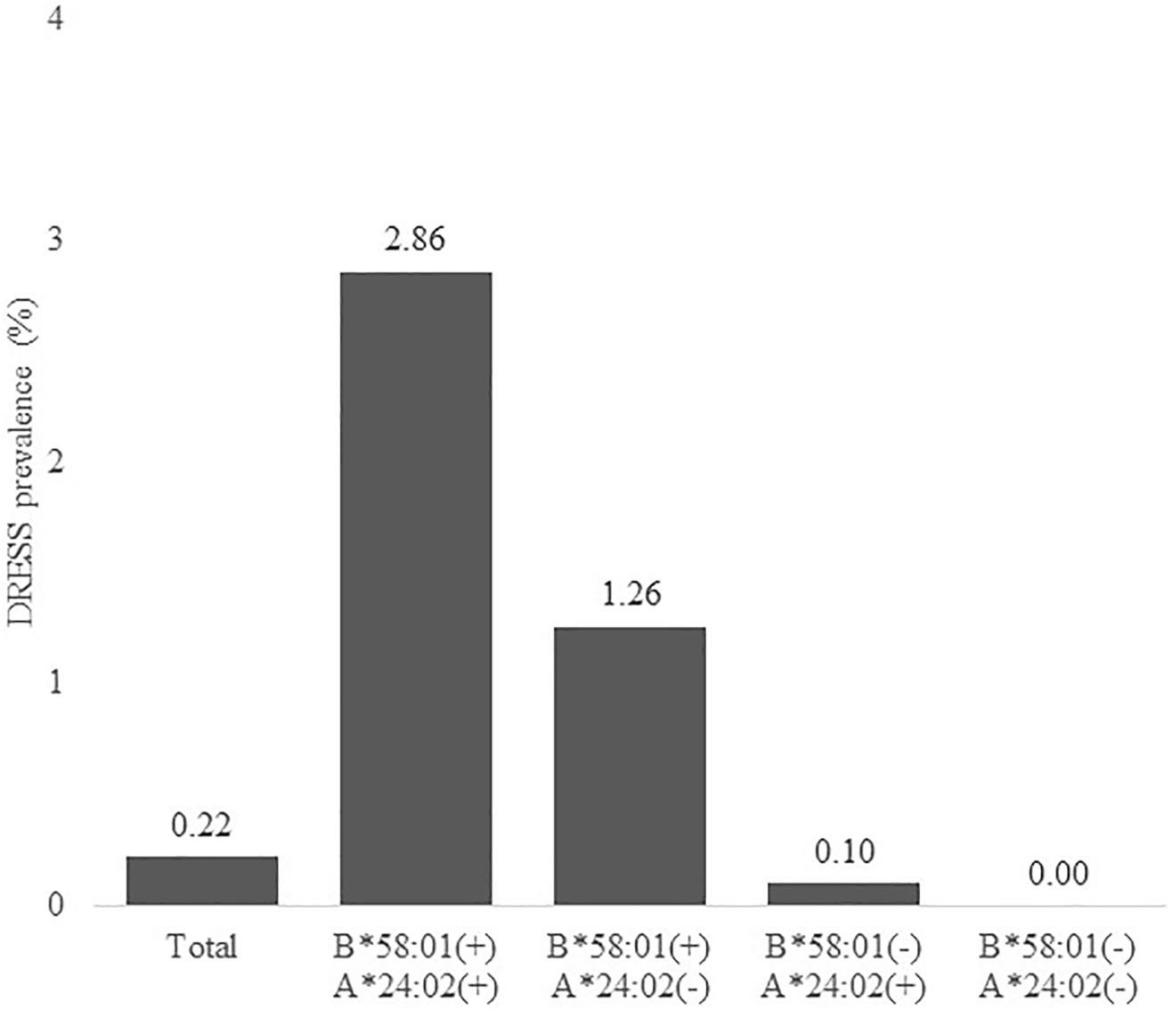
The prevalence of allopurinol‐induced drug reaction with eosinophilia and systemic symptoms (DRESS) according to the HLA phenotype in the HLA‐tested allopurinol user cohort. DRESS, Drug reaction with eosinophilia and systemic symptoms; HLA, human leukocyte antigen

The presence of A*24:02 in B*58:01 carriers with allopurinol‐induced DRESS was assessed in the data of 2782 HLA‐tested allopurinol user cohort. The HLA‐tested allopurinol user cohort included 6 DRESS and the remaining 2776 subjects were considered as the controls. The phenotype frequency of A*24:02 and B*58:01 was significantly higher in the DRESS group than in the controls (33.3% vs. 2.4%, *p* = 0.003, OR = 107.0, 95% CI, 5.1–2250.0) (Table 3). The overall prevalence of DRESS was 0.22% and different according to the presence of B*58:01 and A*24:02 in the HLA‐tested allopurinol user cohort; highest in subjects with both A*24:02 and B*58:01 allele (2.86%), followed by B*58:01 single positive subjects (1.26%), A*24:02 single positive subjects (0.1%), and those without neither of them (0.0%) (Figure 1). The positive predictive value (PPV) and negative predictive value (NPV) of both B*58:01 and A*24:02 screening for the development of allopurinol‐induced DRESS were calculated in the HLA‐tested allopurinol user cohort. Among 309 B*58:01 carriers, 70 subjects had A*24:02 (22.7%) including 2 DRESS. Therefore, PPV and NPV of B*58:01 and A*24:02 screening for DRESS was 2.86% (2/70) and 99.85% (2708/2712), respectively (Table 4). Compared to B*58:01 single screening, B*58:01 and A*24:02 screening improved PPV (from 1.62% to 2.86%) and specificity (from 89.05% to 97.55%), respectively.

**TABLE 1:** Demographics and clinical features of the study subjects

	B*58:01 (+) allopurinol induced DRESS (*n* = 21)	B*58:01 (+) allopurinol induced SJS/TEN (*n* = 6)	B*58:01 (+) allopurinol tolerant control (*n* = 52)
	(%) or median (range)		
Age (years)^a^^,^^b^	57.5 (13–89)	75.5 (27–86)	49.5 (8–72)
Gender (male)^a^	35.0	50.0	71.2
BMI (kg/m^2^)	24.4 (16.4–30.3)	24.0 (20.6–29.4)	22.9 (17.1–38.5)
Cardiovascular disease (%)	55.0	66.7	34.6
Baseline eGFR (ml/min/1.73 m^2^)^a^^,^^b^	38.8 (3.2–132.6)	56.6 (31.7–120.6)	29.0 (2.9–78.0)
Allopurinol maintenance dose (mg/day)^a^^,^^b^	127.8 (100–300)	150 (100–200)	95.5 (25–400)

Abbreviations: BMI, Body mass index; DRESS, Drug reaction with eosinophilia and systemic symptoms; eGFR, estimated Glomerular fractional rate using CKD‐EPI; SJS, Stevens‐Johnson syndrome; TEN, Toxic epidermal necrolysis.

There were significant differences between DRESS versus tolerant (a) and SJS/TEN versus tolerant (b); *p* value < 0.05.

**TABLE 2:** Comparison of HLA phenotype frequency between SJS/TEN, drug reaction with eosinophilia and systemic symptoms (DRESS), allopurinol tolerant controls and general population controls carrying B*58:01 allele

HLA alleles	B*58:01 (+) allopurinol induced DRESS (*n* = 21)	B*58:01 (+) allopurinol induced SJS/TEN (*n* = 6)	B*58:01 (+) allopurinol tolerant controls (*n* = 52)	B*58:01 (+) general population controls^a^ (*n* = 1321)	OR (95% CI), *p* value	
					DRESS versus allopurinol tolerant controls	SJS/TEN versus allopurinol tolerant controls
					DRESS versus general population controls	SJS/TEN versus general population controls
A*24:02 (+)	15 (71.4)	1 (16.7)	9 (17.3)	227 (17.2)	12.0 (3.6–39.2), <0.001	1.0 (0.1–9.2), 1.000
A*24:02 (−)	6 (28.6)	5 (83.3)	43 (82.7)	1094 (82.8)	12.0 (4.6–31.3), <0.001	1.0 (0.1–8.3), 0.973
A*24:02 (+)/DRB1*13:02 (+)	11 (52.4)	0 (0)	3 (5.8)	NA	66.0 (6.1–716.2), <0.001	0.9 (0.7–1.0), 1.000
A*24:02 (−)/DRB1*13:02 (−)	1 (4.8)	3 (50.0)	18 (34.6)	NA	NA	NA

Abbreviations: CI, confidence interval; DRESS, Drug reaction with eosinophilia and systemic symptoms; HLA, Human leukocyte antigen; NA, not assessed; OR, Odds ratio; SJS, Stevens‐Johnson syndrome; TEN, Toxic epidermal necrolysis.

^a^ B*58:01 (+) general population controls means 1321 HLA‐B*58:01 carriers who were extracted from a dataset of 11,998 HLA‐tested patients in a single tertiary hospital. There were not sufficient data of HLA‐DR.

**TABLE 3:** Comparison of HLA phenotype frequency between drug reaction with eosinophilia and systemic symptoms (DRESS) and controls among HLA‐tested allopurinol user cohort (*n* = 2782)

HLA alleles	DRESS (*n* = 6)	Controls (*n* = 2776)	DRESS versus controls
	N (%)	N (%)	OR (95% CI), *p* value
B*58:01 (+) (*n* = 309)	5 (83.3)	304 (11.0)	40.7 (4.7–349.2), <0.001
B*58:01 (−) (*n* = 2473)	1 (16.7)	2472 (89.0)	1
B*58:01 (+) and A*24:02 (+) (*n* = 70)	2 (33.3%)	68 (2.4)	107.0 (5.1–2250.0), 0.003
B*58:01 (+) and A*24:02 (−) (*n* = 239)	3 (50%)	236 (8.5)	43.4 (2.2–842.5), 0.013
B*58:01 (−) and A*24:02 (+) (*n* = 1008)	1 (16.7%)	1007 (36.3)	4.4 (0.2–107.2), 0.367
B*58:01 (−) and A*24:02 (−) (*n* = 1465)	0 (0%)	1465 (52.8)	1

*Note*: HLA‐tested allopurinol user cohort was extracted from a dataset of 11,998 HLA‐tested patients in a single tertiary hospital (unpublished data). All those who did not experience allopurinol‐induced DRESS were considered controls.

Abbreviations: DRESS, Drug reaction with eosinophilia and systemic symptoms; HLA, Human leukocyte antigen.

**TABLE 4:** Calculation of sensitivity, specificity, positive predictive value, and negative value of screening for allopurinol‐induced drug reaction with eosinophilia and systemic symptoms (DRESS) with single HLA‐B*58:01 and a combination of B*58:01 and A*24:02 in the HLA‐tested allopurinol user cohort^a^

	B*58:01	B*58:01/A*24:02
Sensitivity	83.33% (5/6)	33.33% (2/6)
Specificity	89.05% (2472/2776)	97.55% (2708/2776)
PPV	1.62% (5/309)	2.86% (2/70)
NPV	99.96% (2465/2473)	99.85% (2708/2712)

Abbreviations: DRESS, Drug reaction with eosinophilia and systemic symptoms; HLA, Human leukocyte antigen; NPV, negative predictive value; PPV, positive predictive value.

^a^ HLA‐tested allopurinol user cohort was extracted from a dataset of 11,998 HLA‐tested patients in a single tertiary hospital (unpublished data). All those who did not experience allopurinol‐induced DRESS were considered controls.

The data of a previous multicenter case‐control study was reassessed to validate the predominance of A*24:02 in B*58:01 DRESS patients.^12^ B*58:01 were found in 19 out of 20 DRESS patients and 9 of them had A*24:02. Comparing with 1321 B*58:01 (+) general population controls, the phenotype frequency of A*24:02 was significantly higher in the DRESS group than in the general population controls (47.4% vs. 17.2%, *p* = 0.002, OR 4.3, 95% CI, 1.7–10.8).

**FIGURE 2:**
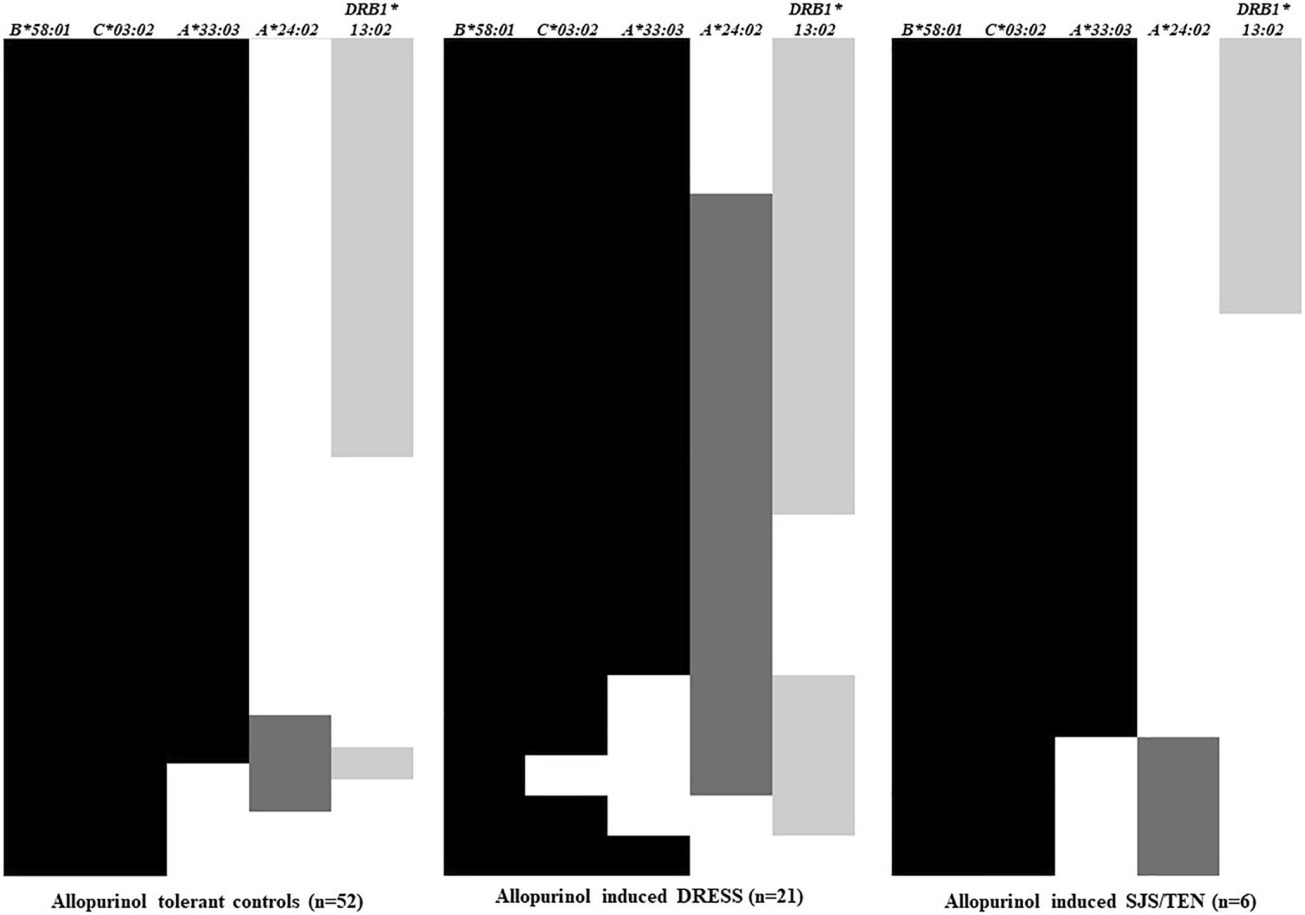
The frequency of A*24:02, and DRB1*13:02 along with putative haplotypes of B*58:01 (C*03:02 and A*33:03) in the allopurinol induced drug reaction with eosinophilia and systemic symptoms (DRESS), SJS/TEN and tolerant controls in B*58:01 carriers in Koreans. DRESS, Drug reaction with eosinophilia and systemic symptoms; HLA, human leukocyte antigen; SJS, Stevens‐Johnson syndrome; TEN, Toxic epidermal necrolysis

There are known linkage disequilibrium in B*58:01‐DRB1*13:02, A*24:02‐C*03:02 and A33:03‐C03:02 in Koreans.^9^ However, A*24:02 still showed higher frequency in DRESS than controls after adjusting A*33:03, C*03:02 and DRB1*13:02 (OR, 19.8; 95% CI, 4.6–85.7, *p* < 0.001). Furthermore, the co‐existence with A*24:02 and DRB1*13:02 showed significantly higher frequency in DRESS than controls after adjusting A*33:03 and C*03:02 (OR, 69.9, 95% CI, 5.8–848.2, *p* = 0.001). In contrast, there was no case of co‐existence of A*24*02 and DRB1*13:02 in the tolerant controls and SJS/TEN group (Figure 2).

### 3.5 Binary logistic regression to evaluate risk factors in considering clinical differences between B*58:01 (+) allopurinol‐induced DRESS and tolerant controls

Binary logistic regression analysis was performed to evaluate HLAs as independent risk factor in this study group (Table S3). First, univariate logistic regression analysis was performed using age (≥60 years old), gender (female), BMI (≥25 kg/m^2^), baseline eGFR (<60 ml/min/1.73 m^2^), maintenance dose of allopurinol (≥200 mg/day), cardiovascular disease (presence or not), A*24:02 (carrier or not) and A*24:02 with DRB1*13:02 (carrier both or none). Between DRESS and control groups, there were differences in gender, eGFR, dose of allopurinol, A*24:02 and A*24:02 with DRB1*13:02 with *p* < 0.1. Multivariate logistic regression analysis with gender, eGFR, dose of allopurinol and A*24:02 was performed. A*24:02 was an independent risk factor in the development allopurinol‐induced DRESS (OR, 55.0; 95% CI, 4.3–702.0; *p* = 0.002). Female, high eGFR (≥60 ml/min/1.73 m^2^) and high dose of allopurinol (≥200 mg/day) were also independent risk factors in the development allopurinol‐induced DRESS.

### 3.6 Comparison of the HLA phenotype frequency of SJS/TEN to allopurinol‐tolerant controls and HLA‐B*58:01 (+) general population controls

No additional HLA alleles increase the risk of allopurinol‐induced SJS/TEN in the B*58:01 carriers (Table 2). Multivariate logistic regression analysis with age, eGFR, dose of allopurinol, and A*24:02 was performed and no significant factor was identified.

## 4 DISCUSSION

We investigated whether subsidiary HLA alleles in addition to B*58:01 could increase the predictability of allopurinol‐induced SCARs and found that the risk of allopurinol‐induced DRESS was higher in B*58:01 carriers if they also had A*24:02. In addition, the presence of DRB1*13:02 in addition to A*24:02 further increased risk of DRESS in B*58:01 carriers. After adjusting with HLAs which were known to have linkage disequilibrium with B*58:01 and clinical factors, A*24:02 was an independent risk factor of allopurinol‐induced DRESS in B*58:01 carriers. In the previous studies,^4^, ^12^, ^13^, ^14^ weaker HLA associations such as A*24:02 and DRB1*13:02 could have been missed as B*58:01 provides much stronger signal. However, by comparing only B*58:01 carriers, we were able to find additional signals.

Various factors may be involved in the binding of drugs between HLA and T cells.^18^ Structural factors for drug recognition include the T cell receptors and major histocompatibility complex (MHC) molecules of antigen presenting cells which present peptides processed from exogenous antigens or endogenous peptides. HLA is a gene complex encoding the MHC molecules in humans and is the most studied risk factor in the development of SCARs.^8^, ^18^, ^19^ In addition, other secondary signals may also be involved in this process.^8^, ^20^ An altered peptide repertoire due to the drug binding to HLA was proposed recently as an alternative mechanism for T cell reactivity in drug hypersensitivity.^21^ Crystallographic structures revealed that certain drug could be embedded underneath the altered peptides and is able to form stable peptide–drug–HLA complexes. Also, it is hypothesized that a small molecule drug like allopurinol can bind to various HLA sites and can induce adverse reactions in various mechanism in addition to direct TCR binding.^22^ A noncovalent interaction of the drug with the HLA molecule that alters the presented peptide‐HLA complex on the surface is likely responsible for allopurinol specific T cell responses.^23^ However, it is not known what role A*24:02 plays in these mechanisms especially when the previously reported molecular docking of allopurinol or oxypurinol on A*24:02 did not reveal high affinity interactions compared to other alleles.^23^

B*58:01 is a well‐known risk factor for the development of allopurinol‐induced SCARs in Asian descendants.^8^ The risk of SCAR development is affected by ethnicity because the allele frequencies are different in ethnic groups.^19^, ^20^ In Koreans, the association between B*58:01 and allopurinol‐induced SCARs was first reported in 2011.^12^ In comparing 25 allopurinol‐induced SCAR patients (20 drug‐induced hypersensitivity syndrome and 5 SJS/TEN) and 57 tolerant with chronic renal failure taking allopurinol for at least 6 months, the frequencies of B*58:01 was significantly different (92.0% vs. 10.5%, OR 97.8, Pc = 2.45 × 10^−11^).^12^ Although the association between B*58:01 and allopurinol‐induced SCAR was confirmed repeatedly, Jung et al. reported that only 18% of CRI patients with B*58:01 had SCAR after taking allopurinol.^13^ This relatively low PPV suggests that B*58:01 is a strong risk factor but B*58:01 is not solely responsible for the development of allopurinol‐induced SCARs. If HLA has an important role in the immunologic mechanism of allopurinol‐induced SCARs, it is likely that other supplementary HLA alleles may play a role, especially in the patient who eventually develop SCARs among B*58:01 carriers. In this study, we found that concomitant screening of A*24:02 and DRB1*13:02 could improve the predictability of allopurinol‐induced DRESS development.

Shim et al. reported that the secondary serotype screening of additional HLA risk factors for B*58:01 carriers could increase the accuracy of the prediction of allopurinol‐induced SCARs and suggested that B75, DR13 homozygosity, and DR14 could be risk factors in Koreans.^4^ B75 is an HLA‐B serotype and includes B*15:02, B*15:08, B*15:11 and B*15:21.^24^ Alleles representing the B75 serotype in Koreans are B*15:02 and B*15:11 with a phenotype frequency of 0.4% and 3.8%, respectively.^9^ However, this finding could not be replicated because no subjects with those alleles were found in the current study. DRB1*13:02 is the only HLA allele belonging to DR13 in this study population. DRB1*13:02 homozygosity was more frequent in DRESS compared to the tolerant controls in the B*58:01 carriers in the current study (DRESS vs. control, 9.5% vs. 3.8%), but the difference was not statistically significant due to the limited numbers (*p* = 0.574). On the other hand, we found that DRB1*13:02 is an additional risk factor in combination with A*24:02, and this points toward a comparable finding. It is difficult to determine which HLA alleles correspond to the serotype DR14 because DR14 is diverse.^9^ In this study, there was only a small number of subjects with DR14 and subtypes were not consistent (two DRB1*14:01, two DRB1*14:05 tolerant controls and one DRB1*14:05 DRESS patient) and there was no significant difference in the phenotype frequency of DR14 between DRESS and tolerant controls (DRESS vs. control, 4.8% vs. 7.7%, *p* = 1.000). The previous study was a retrospective cohort study of B*58:01 carriers and hence, included a relatively large number of allopurinol tolerant controls (*n* = 109) but a small number of SCAR patients (*n* = 11).^4^ On the other hand, our current case‐control study had a limited number of allopurinol tolerant controls but included a larger number of SCAR patients, and this may explain different findings. The validation study is difficult in SCAR due to its rarity. To prove the significance of A*24:02, we successfully replicated our finding in two separate populations. In the previous report, A*24:02 predominance could not be recognized because there were only six B*58:01 carrier among 57 subjects in the allopurinol tolerant group. Considering the consistent findings in replication studies and high specificity of B*58:01/A*24:02 in predicting the risk of DRESS development, A*24:02 could be applied as a secondary HLA testing in those who are B*58:01 positive.

The role that A*24:02 and DRB1*13:02 may play in the pathogenesis of allopurinol‐induced DRESS is currently unknown. Interestingly, A*24:02 was shown to be an additional risk factor for the development of carbamazepine‐induced SJS/TEN in Han Chinese and was also reported to be associated with SJS/TEN related with NSAID and cold medication in Japanese.^25^, ^26^ However, there was no reported association of A*24:02 with SCARs in Korea. There are reports that some HLA class II molecules were associated with viral infection, such as infectious mononucleosis, and with neuroinflammatory activity and progression in patients with chronic hepatitis C.^27^, ^28^ Although DRB1*13:02 alone did not increase the risk of DRESS development in our study, it could have some facilitating roles in the development of DRESS because virus reactivations are involved in the pathogenesis of DRESS.^29^ It is plausible that MHC class II molecules may play a role in the immune response as a report showed that DRB1 alleles influence Th1 and Th2 cytokine responses to *Mycobacterium tuberculosis* antigens.^30^ Therefore, the increased risk by the presence of DRB1*13:02 raised the possibility that the binding between allopurinol and certain MHC class II may help to activate Th2 inflammation.

Our study suggests the possibility of differences in the genetic susceptibility of SJS/TEN and DRESS. Three HLA alleles, A*02:06, C*04:01, and DRB1*04:06, were higher in SJS/TEN while two HLA alleles, A*24:02 and DRB1*13:02, were more frequent in DRESS. There was known haplotype frequency between B*58:01 and DRB1*13:02 in general Korean population (3.29%).^9^ However, there was no linkage between A*02:06, C*04:01, B*58:01 and DRB1*04:06 in the general Korean population.^9^ Genetic susceptibilities limited to specific phenotypes of SCARs are already described in the studies on carbamazepine SCARs; A*31:01 is associated with DRESS while B*15:02 and B*15:11 are associated with SJS/TEN.^25^, ^31^ Because B*58:01 is a strong risk factor associated with both phenotypes of SCARs induced by allopurinol, it may have been difficult to discover the minor risk factors contributing to the development of specific phenotypes. This study reported, for the first time, the possibility of HLA differences between allopurinol‐induced SJS/TEN and DRESS. However, it was not possible to draw a firm conclusion because the number of SJS/TEN patients in this study was not large enough. SCARs sometimes result in drug‐induced hypersensitivity syndrome/TEN overlap, encompassing the clinical features of both phenotypes.^32^ The results of this study suggest that it is important to analyse SCAR according to its phenotypes.

In this study, there are some limitations. First, since we obtained the allopurinol control group from pre‐tested HLA subjects, relatively severe CRI patients were included to the tolerant control. These subjects might be regarded as a specific disease group, and they are frequently exposed to allopurinol because of renal impairment. Considering that severe renal impairment is one of the key risk factors for the SCAR development, it is an important factor in the analysis of the tolerability to allopurinol. However, A*24:02 was shown to be an independent risk factor for the development of allopurinol‐induced DRESS in B*58:01 carriers on multivariate logistic regression analysis including impaired renal function. Secondly, since we did not sequence the whole HLA, the haplotype information was inferred but not confirmed. However, considering previous literature, B*58:01 has strong linkage disequilibrium with A*33:03 in Koreans. In our data, phenotype frequency of A*33:03 was 84.8% among B*58:01 carriers. Therefore, it is unlikely that linkage disequilibrium exists between A*24:02 and B*58:01. Finally, since this study was done in a Korean population, replication studies need to be performed before the findings can be generalized, considering the ethnic differences exist in HLA distributions.

In conclusions, additional secondary screening with A*24:02 and DRB1*13:02 may be implemented in a clinical setting to further evaluate the risk of developing DRESS in B*58:01 carriers taking allopurinol. Prospective studies should be performed in the future to validate this finding.

**Abbreviations**
BMI: body mass index
CKD‐EPI: Chronic Kidney Disease Epidemiology Collaboration
CRI: chronic renal impairment
DRESS: drug reaction with eosinophilia and systemic symptoms
eGFR: estimated glomerular fractional rate
EULAR: The European League Against Rheumatism
HLA: human leukocyte antigen
IRB: Institutional Review Board
MHC: major histocompatibility complex
SCAR: severe cutaneous adverse reaction
SJS: Stevens‐Johnson syndrome
TEN: toxic epidermal necrolysis
WHO: World Health Organization

## AUTHOR CONTRIBUTIONS

Mi‐Yeong Kim: Conceptualization (lead); Data curation (equal); Formal analysis; Equal, Funding acquisition (equal); Investigation (equal); Methodology (equal); Project administration (lead); Validation (equal); Visualization (equal); Writing – original draft (lead); Writing – review & editing (equal). Hye‐Ryun Kang: Conceptualization (lead); Data curation (lead); Formal analysis (equal); Funding acquisition (equal); Investigation (lead); Methodology (lead); Project administration (equal); Resources (equal); Supervision (lead); Validation (equal); Visualization (equal); Writing – review & editing (lead). James Yun: Writing – review & editing (supporting). Dong Yoon Kang: Methodology (supporting). Tae Hee Kim: Data curation (equal). Min‐Kyung Oh: Methodology (supporting). Sunggun Lee: Data curation (equal). Min‐Gyu Kang: Data curation (equal). Young‐Hee Nam: Data curation (equal). Jeong‐Hee Choi: Data curation (equal). Min‐Suk Yang: Data curation (equal). Seung Seok Han: Data curation (equal). Hajeong Lee: Data curation (Equal). Hyun‐Jai Cho: Data curation (equal). Jaeseok Yang: Data curation (equal). Kook‐Hwan Oh: Data curation (equal). Yon Su Kim: Data curation (equal). Kye Hwa Lee: Data curation (equal); Investigation (equal); Validation (equal). Jae‐Woo Jung: Data curation (equal).

## CONFLICT OF INTEREST

The authors declare that they have no competing interests.

## ETHNICS STATEMENT

This study was approved by the Institutional Review Board (IRB) of Seoul National University Hospital (IRB No: H‐1805‐060‐945) and informed consent was waived by the IRB of Inje University Busan Paik Hospital (IRB No: 18–0071) for the use of samples and clinical informations in the Inje Biobank.

## CONSENT FOR PUBLICATION

Not applicable.

## Supporting information

Supporting Information S1

## Data Availability

The authors confirm that the main data supporting the findings of this study are available within the article and its supplementary materials. The supporting data used for the validation sets are also available within the original articles.

## References

[1] Richette P , Doherty M , Pascual E , et al. EULAR evidence‐based recommendations for the management of gout updated. Ann Rheum Dis. 2016;76(1):29‐42. 10.1136/annrheumdis-2016-209707 27457514

[2] Elion GB , Yu TF , Gutman AB , Hitchings GH . Renal clearance of oxipurinol, the chief metabolite of allopurino. Am J Med. 1968;45(1):69‐77. 10.1016/0002-9343(68)90008-9 5658870

[3] Stamp LK , Chapman PT , Barclay M , et al. The effect of kidney function on the urate lowering effect and safety of increasing allopurinol above doses based on creatinine clearance: a post hoc analysis of a randomized controlled trial. Arthritis Res Ther. 2017;19(1):283. 10.1186/s13075-017-1491-x 29268756 PMC5740867

[4] Shim JS , Yun J , Kim MY , et al. The presence of HLA‐B75, DR13 homozygosity, or DR14 additionally increases the risk of allopurinol‐induced severe cutaneous adverse reactions in HLA‐B*58:01 carriers. J Allergy Clin Immunol Pract. 2019;7(4):1261‐1270. 10.1016/j.jaip.2018.11.039 30529060

[5] White WB , Saag KG , Becker MA , et al. Gunawardhana L, investigators C, cardiovascular safety of febuxostat or allopurinol in patients with gout. N Engl J Med. 2018;378(13):1200‐1210. 10.1056/NEJMoa1710895 29527974

[6] Stamp LK , Day RO , Yun J . Allopurinol hypersensitivity: investigating the cause and minimizing the risk. Nat Rev Rheumatol. 2016;12(4):235‐242. 10.1038/nrrheum.2015.132 26416594

[7] Ramasamy SN , Korb‐Wells CS , Kannangara DR , et al. Allopurinol hypersensitivity: a systematic review of all published cases, 1950‐2012. Drug Saf 2013;36(10):953‐980. 10.1007/s40264-013-0084-0 23873481

[8] Wang CW , Dao RL , Chung WH . Immunopathogenesis and risk factors for allopurinol severe cutaneous adverse reactions. Curr Opin Allergy Clin Immunol. 2016;16(4):339‐345. 10.1097/ACI.0000000000000286 27362322

[9] Lee KW , Oh DH , Lee C , Yang SY . Allelic and haplotypic diversity of HLA‐A, ‐B, ‐C, ‐DRB1, and ‐DQB1 genes in the Korean population. Tissue Antigens. 2005;65(5):437‐447. 10.1111/j.1399-0039.2005.00386.x 15853898

[10] Khan DA . Pharmacogenomics and adverse drug reactions: primetime and not ready for primetime tests. J Allergy Clin Immunol. 2016;138(4):943‐955. 10.1016/j.jaci.2016.08.002 27720019

[11] Kim M.‐Y , Yang M.‐S , Kang H.‐R , Cho S.‐H , Min K.‐U . Analysis of drugs causing severe cutaneous adverse reactions, based on the Korean Database of spontaneously reported adverse drug reactions. Korean J Med. 2014;86(6):710. 10.3904/kjm.2014.86.6.710

[12] Kang HR , Jee YK , Kim YS , et al. Adverse drug reaction research group in korea, positive and negative associations of HLA class I alleles with allopurinol‐induced SCARs in koreans. Pharmacogenet Genom; 2011. 10.1097/FPC.0b013e32834282b8 21301380

[13] Jung JW , Song WJ , Kim YS , et al. HLA‐B58 can help the clinical decision on starting allopurinol in patients with chronic renal insufficiency. Nephrol Dial Transpl. 2011;26(11):3567‐3572. 10.1093/ndt/gfr060 21393610

[14] Jung JW , Kim DK , Park HW , et al. An effective strategy to prevent allopurinol‐induced hypersensitivity by HLA typing. Genet Med. 2015;17(10):807‐814. 10.1038/gim.2014.195 25634024

[15] Kang DY , Yun J , Lee SY , et al. Nationwide study of severe cutaneous adverse reactions based on the multicenter registry in Korea. J Allergy Clin Immunol Pract. 2021;9(2):929‐936.e7. 10.1016/j.jaip.2020.09.011 32961314

[16] Rehan HS , Chopra D , Kakkar AK . Physician's guide to pharmacovigilance: terminology and causality assessment. Eur J Intern Med. 2009;20(1):3‐8. 10.1016/j.ejim.2008.04.019 19237084

[17] Lee KH , Kang DY , Kim HH , et al. Reducing severe cutaneous adverse and type B adverse drug reactions using pre‐stored human leukocyte antigen genotypes. Clin Transl Allergy. 2022;12(1). 10.1002/clt2.12098 PMC8760506 35070271

[18] Pavlos R , Mallal S , Ostrov D , et al. T cell‐mediated hypersensitivity reactions to drugs. Annu Rev Med. 2015;66(1):439‐454. 10.1146/annurev-med-050913-022745 25386935 PMC4295772

[19] Pavlos R , Mallal S , Phillips E . HLA and pharmacogenetics of drug hypersensitivity. Pharmacogenomics. 2012;13(11):1285‐1306. 10.2217/pgs.12.108 22920398

[20] Pichler WJ , Naisbitt DJ , Park BK . Immune pathomechanism of drug hypersensitivity reactions. J Allergy Clin Immunol. 2011;127(3):S74‐S81. 10.1016/j.jaci.2010.11.048 21354503

[21] Illing PT , Vivian JP , Dudek NL , et al. Immune self‐reactivity triggered by drug‐modified HLA‐peptide repertoire. Nature. 2012;486(7404):554‐558. 10.1038/nature1147 22722860

[22] Pompeu YA , Stewart JD , Mallal S , Phillips E , Peters B , Ostrov DA . The structural basis of HLA‐associated drug hypersensitivity syndromes. Immunol Rev. 2012;250(1):158‐166. 10.1111/j.1600-065x.2012.01163.x 23046128

[23] Yun J , Marcaida MJ , Eriksson KK , et al. Oxypurinol directly and immediately activates the drug‐specific T cells via the preferential use of HLA‐B*58:01. J Immunol. 2014;192(7):2984‐2993. 10.4049/jimmunol.1302306 24591375

[24] Hwang SH , Oh HB , Yang JH , Kwon OJ , Shin ES . Distribution of HLA‐A, B, C allele and haplotype frequencies in Korean. Korean J Lab Med. 2004;24(6):396‐404.

[25] Hung SI , Chung WH , Jee SH , et al. Genetic susceptibility to carbamazepine‐induced cutaneous adverse drug reactions. Pharmacogenet Genom 2006;16(4):297‐306. 10.1097/01.fpc.0000199500.46842.4a 16538176

[26] Ueta M , Kaniwa N , Sotozono C , et al. Independent strong association of HLA‐A*02:06 and HLA‐B*44:03 with cold medicine‐related Stevens‐Johnson syndrome with severe mucosal involvement. Sci Rep. 2014;4(1):4862. 10.1038/srep04862 24781922 PMC5381277

[27] Ramagopalan SV , Meier UC , Conacher M , et al. Role of the HLA system in the association between multiple sclerosis and infectious mononucleosis. Arch Neurol. 2011;68(4):469. 10.1001/archneurol.2011.48 21482926

[28] Kryczka W , Brojer E , Kalinska A , Urbaniak A , Zarebska‐Michaluk D . DRB1 alleles in relation to severity of liver disease in patients with chronic hepatitis C. Med Sci Mon Int Med J Exp Clin Res. 2001;7(Suppl 1):217‐220. 12211723

[29] Shiohara T , Ushigome Y , Kano Y , Takahashi R . Crucial role of viral reactivation in the development of severe drug eruptions: a comprehensive review. Clin Rev Allergy Immunol. 2015;49(2):192‐202. 10.1007/s12016-014-8421-3 24736996

[30] Selvaraj P , Nisha Rajeswari D , Jawahar MS , Narayanan PR . Influence of HLA‐DRB1 alleles on Th1 and Th2 cytokine response to Mycobacterium tuberculosis antigens in pulmonary tuberculosis. Tuberculosis. 2007;87(6):544‐550. 10.1016/j.tube.2007.08.001 17826339

[31] Phillips EJ , Mallal SA . Pharmacogenetics of drug hypersensitivity. Pharmacogenomics. 2010;11(7):973‐987. 10.2217/pgs.10.77 20602616 PMC2937181

[32] Bouvresse S , Valeyrie‐Allanore L , Ortonne N , et al. Toxic epidermal necrolysis, DRESS, AGEP: do overlap cases exist? Orphanet J Rare Dis. 2012;7(1):72. 10.1186/1750-1172-7-72 23009177 PMC3517389

