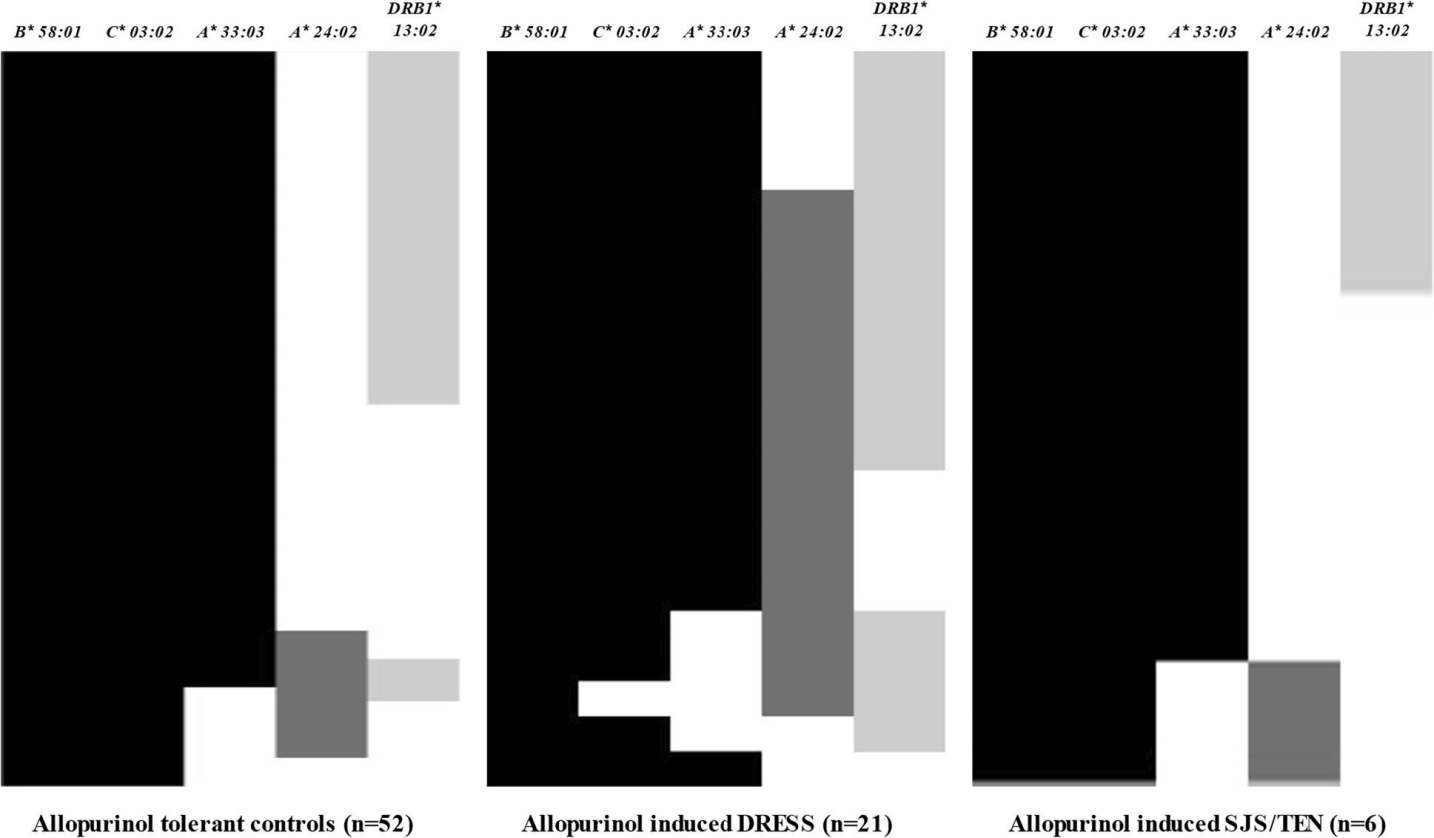# Correction to HLA‐A*24:02 increase the risk of allopurinol‐induced drug reaction with eosinophilia and systemic symptoms (DRESS) in HLA‐B*58:01 carriers in Korean population; A multicenter cross‐sectional case‐control study

**DOI:** 10.1002/clt2.12351

**Published:** 2024-04-05

M. Y. Kim, J. Yun, D. Y. Kang, T. H. Kim, M. K. Oh, S. Lee, et al. HLA‐A* 24: 02 increase the risk of allopurinol‐induced drug reaction with eosinophilia and systemic symptoms in HLA‐B* 58: 01 carriers in a Korean population; a multicenter cross‐sectional case‐control study. Clin Transl Allergy. 2022 Sep 15;12(9):e12193.

This article [1] was published with a technical error in Figure 2, a size of bars in the allopurinol‐tolerant controls. The authors have re‐examined the data and confirmed that this correction would not have resulted in any alteration to the results or conclusion of the paper. The figure 2 should be shown as below.