# Supplementary material for: HLA‐A*24:02 increase the risk of allopurinol‐induced drug reaction with eosinophilia and systemic symptoms in HLA‐B*58:01 carriers in a Korean population; a multicenter cross‐sectional case‐control study
Source: Clin Transl Allergy. 2022 Sep 15;12(9):e12193. doi: 10.1002/clt2.12193 (PMC9478421; doi:10.1002/clt2.12193)
Supplement: Supplementary file 1 — Supporting Information S1 [file CLT2-12-e12193-s001.docx]

Table S1. Results of HLA-A, B, C, and DRB1 alleles analysis in the subjects with B*58:01

| Group | Number | HLA-A* | | HLA-B* | | HLA-C* | | HLA-DRB1* | |
| --- | --- | --- | --- | --- | --- | --- | --- | --- | --- |
| Tolerant control | 1 | 26:01 | 31:01 | 51:02 | 58:01 | 03:02 | 15:02 | 04:05 | 16:02 |
|  | 2 | 33:03 | 33:03 | 58:01 | 58:01 | 03:02 | 03:02 | 03:01 | 13:02 |
|  | 3 | 02:01 | 33:03 | 40:01 | 58:01 | 03:02 | 15:02 | 13:02 | 15:01 |
|  | 4 | 24:02 | 26:03 | 35:01 | 58:01 | 03:02 | 07:02 | 13:02 | 14:05 |
|  | 5 | 11:01 | 33:03 | 15:01 | 58:01 | 03:02 | 04:01 | 04:06 | 13:02 |
|  | 6 | 29:01 | 33:03 | 07:05 | 58:01 | 03:02 | 15:05 | 08:03 | 13:02 |
|  | 7 | 02:07 | 33:03 | 35:01 | 58:01 | 03:02 | 03:03 | 09:01 | 13:02 |
|  | 8 | 24:02 | 33:03 | 54:01 | 58:01 | 01:02 | 03:02 | 04:05 | 14:01 |
|  | 9 | 11:01 | 33:03 | 15:01 | 58:01 | 01:02 | 03:02 | 03:01 | 04:03 |
|  | 10 | 02:01 | 33:03 | 46:01 | 58:01 | 03:02 | 04:01 | 03:01 | 08:03 |
|  | 11 | 31:01 | 33:03 | 55:02 | 58:01 | 03:02 | 03:03 | 08:03 | 13:02 |
|  | 12 | 24:02 | 33:03 | 46:01 | 58:01 | 01:02 | 03:02 | 03:01 | 15:01 |
|  | 13 | 11:01 | 33:03 | 54:01 | 58:01 | 01:02 | 03:02 | 10:01 | 13:02 |
|  | 14 | 11:01 | 24:02 | 46:01 | 58:01 | 01:02 | 03:02 | 03:01 | 08:03 |
|  | 15 | 02:07 | 33:03 | 46:01 | 58:01 | 01:02 | 03:02 | 04:10 | 09:01 |
|  | 16 | 02:06 | 33:03 | 15:01 | 58:01 | 03:02 | 04:01 | 03:01 | 09:01 |
|  | 17 | 33:03 | 33:03 | 44:03 | 58:01 | 03:02 | 14:03 | 12:01 | 13:02 |
|  | 18 | 11:01 | 24:02 | 15:01 | 58:01 | 03:02 | 04:01 | 04:05 | 04:06 |
|  | 19 | 33:03 | 33:03 | 13:02 | 58:01 | 03:02 | 06:02 | 08:03 | 13:02 |
|  | 20 | 31:01 | 33:03 | 51:01 | 58:01 | 03:02 | 14:02 | 13:02 | 16:02 |
|  | 21 | 02:01 | 33:03 | 15:18 | 58:01 | 03:02 | 07:04 | 09:01 | 13:02 |
|  | 22 | 02:06 | 11:01 | 46:01 | 58:01 | 01:03 | 03:02 | 03:01 | 11:01 |
|  | 23 | 33:03 | 33:03 | 51:01 | 58:01 | 03:02 | 03:02 | 03:01 | 12:02 |
|  | 24 | 02:06 | 33:03 | 35:01 | 58:01 | 03:02 | 03:03 | 03:01 | 09:01 |
|  | 25 | 33:03 | 33:03 | 44:03 | 58:01 | 03:02 | 14:03 | 13:02 | 13:02 |
|  | 26 | 11:01 | 33:03 | 39:01 | 58:01 | 03:02 | 07:02 | 03:01 | 08:03 |
|  | 27 | 02:01 | 33:03 | 40:02 | 58:01 | 03:02 | 03:04 | 03:01 | 11:01 |
|  | 28 | 33:03 | 33:03 | 58:01 | 58:01 | 03:02 | 03:02 | 13:02 | 15:01 |
|  | 29 | 02:06 | 33:03 | 40:02 | 58:01 | 03:02 | 03:04 | 04:05 | 13:02 |
|  | 30 | 11:01 | 33:03 | 39:01 | 58:01 | 03:02 | 07:02 | 01:01 | 08:03 |
|  | 31 | 11:01 | 33:03 | 44:03 | 58:01 | 03:02 | 07:01 | 07:01 | 13:02 |
|  | 32 | 24:02 | 33:03 | 51:01 | 58:01 | 03:02 | 15:02 | 13:02 | 14:05 |
|  | 33 | 33:03 | 33:03 | 44:03 | 58:01 | 03:02 | 14:03 | 03:01 | 13:02 |
|  | 34 | 33:03 | 33:03 | 44:03 | 58:01 | 03:02 | 07:01 | 07:01 | 13:02 |
|  | 35 | 02:07 | 33:03 | 15:01 | 58:01 | 03:02 | 04:01 | 03:01 | 04:06 |
|  | 36 | 26:03 | 33:03 | 35:01 | 58:01 | 03:02 | 03:03 | 12:01 | 15:01 |
|  | 37 | 33:03 | 33:03 | 58:01 | 58:01 | 03:02 | 03:02 | 12:01 | 13:02 |
|  | 38 | 02:01 | 33:03 | 54:01 | 58:01 | 01:02 | 03:02 | 04:05 | 13:02 |
|  | 39 | 11:01 | 33:03 | 44:03 | 58:01 | 03:02 | 14:03 | 03:01 | 10:01 |
|  | 40 | 03:01 | 33:03 | 35:01 | 58:01 | 03:02 | 03:03 | 11:01 | 15:01 |
|  | 41 | 11:01 | 33:03 | 15:01 | 58:01 | 03:02 | 04:01 | 03:01 | 16:02 |
|  | 42 | 02:06 | 33:03 | 48:01 | 58:01 | 03:02 | 03:03 | 12:02 | 13:02 |
|  | 43 | 30:04 | 33:03 | 14:01 | 58:01 | 03:02 | 08:02 | 08:02 | 13:02 |
|  | 44 | 02:06 | 33:03 | 54:01 | 58:01 | 03:02 | 01:02 | 04:05 | 13:02 |
|  | 45 | 24:02 | 02:07 | 15:01 | 58:01 | 03:02 | 04:01 | 03:01 | 04:06 |
|  | 46 | 24:08 | 33:03 | 35:01 | 58:01 | 03:02 | 03:04 | 13:02 | 13:02 |
|  | 47 | 11:01 | 33:03 | 15:01 | 58:01 | 03:02 | 01:02 | 03:01 | 04:03 |
|  | 48 | 31:01 | 33:03 | 15:01 | 58:01 | 03:02 | 03:03 | 12:02 | 14:01 |
|  | 49 | 02:01 | 33:03 | 40:01 | 58:01 | 03:02 | 15:02 | 08:03 | 13:02 |
|  | 50 | 11:01 | 33:03 | 54:01 | 58:01 | 03:02 | 01:02 | 08:03 | 13:02 |
|  | 51 | 24:02 | 02:01 | 40:01 | 58:01 | 03:02 | 03:04 | 03:01 | 12:01 |
|  | 52 | 24:02 | 33:03 | 52:01 | 58:01 | 03:02 | 12:02 | 15:02 | 13:02 |
| DRESS | 1 | 24:02 | 33:03 | 58:01 | 59:01 | 01:02 | 03:02 | 04:05 | 13:02 |
|  | 2 | 24:02 | 33:03 | 44:03 | 58:01 | 03:02 | 14:03 | 13:02 | 13:02 |
|  | 3 | 30:01 | 33:03 | 13:02 | 58:01 | 03:02 | 06:02 | 09:01 | 13:02 |
|  | 4 | 24:02 | 33:03 | 58:01 | 35:01 | 01:02 | 03:02 | 03:01 | 15:01 |
|  | 5 | 24:02 | 33:03 | 51:01 | 58:01 | 03:02 | 15:02 | 04:03 | 13:02 |
|  | 6 | 02:01 | 33:03 | 15:01 | 58:01 | 03:02 | 04:01 | 04:03 | 13:02 |
|  | 7 | 33:03 | 24:02 | 58:01 | 40:06 | 03:02 | 03:03 | 12:01 | 13:02 |
|  | 8 | 24:02 | 33:03 | 07:02 | 58:01 | 03:02 | 07:02 | 01:01 | 13:02 |
|  | 9 | 24:02 | 24:02 | 58:01 | 59:01 | 01:02 | 03:02 | 04:05 | 13:02 |
|  | 10 | 24:02 | 33:03 | 51:01 | 58:01 | 03:02 | 14:02 | 03:01 | 09:01 |
|  | 11 | 02:01 | 33:03 | 15:01 | 58:01 | 03:02 | 03:03 | 09:01 | 14:05 |
|  | 12 | 11:01 | 24:02 | 40:01 | 58:01 | 03:04 | 07:01 | 04:03 | 13:02 |
|  | 13 | 02:01 | 33:03 | 58:01 | 58:01 | 03:02 | 03:02 | 13:02 | 13:02 |
|  | 14 | 24:02 | 33:03 | 48:01 | 58:01 | 03:02 | 08:03 | 13:02 | 15:01 |
|  | 15 | 24:02 | 33:03 | 40:06 | 58:01 | 03:02 | 08:01 | 09:01 | 13:02 |
|  | 16 | 24:02 | 33:03 | 07:02 | 58:01 | 03:02 | 07:02 | 01:01 | 15:01 |
|  | 17 | 02:01 | 33:03 | 40:02 | 58:01 | 03:02 | 03:04 | 08:02 | 13:02 |
|  | 18 | 24:02 | 33:03 | 40:01 | 58:01 | 03:02 | 03:03 | 09:01 | 15:01 |
|  | 19 | 02:07 | 31:01 | 46:01 | 58:01 | 01:02 | 03:02 | 08:03 | 13:02 |
|  | 20 | 30:04 | 24:02 | 58:01 | 39:01 | 03:02 | 07:02 | 08:03 | 13:02 |
|  | 21 | 24:02 | 33:03 | 40:02 | 58:01 | 03:02 | 03:03 | 11:01 | 13:02 |
| SJS/TEN | 1 | 02:01 | 33:03 | 40:01 | 58:01 | 03:02 | 15:02 | 01:01 | 04:05 |
|  | 2 | 02:07 | 33:03 | 46:01 | 58:01 | 01:02 | 03:02 | 09:01 | 12:02 |
|  | 3 | 02:06 | 33:03 | 15:01 | 58:01 | 03:02 | 04:01 | 04:06 | 13:02 |
|  | 4 | 33:03 | 33:03 | 40:02 | 58:01 | 03:02 | 03:04 | 03:01 | 09:01 |
|  | 5 | 11:01 | 33:03 | 15:01 | 58:01 | 03:02 | 04:01 | 04:06 | 13:02 |
|  | 6 | 02:06 | 24:02 | 40:01 | 58:01 | 03:02 | 04:01 | 04:01 | 08:03 |

Abbreviation

HLA, Human leukocyte antigen; DRESS, Drug reaction with eosinophilia and systemic symptoms; SJS, Stevens-Johnson syndrome; TEN, Toxic epidermal necrolysis

Table S2. Phenotype frequency of HLA-A, B, C, and DRB1 alleles in the subjects with B*58:01 carriers

|  | Allopurinol  tolerant control*  (n=52) | | Allopurinol  induced SCAR  (n=27) | | Allopurinol  induced DRESS  (n=21) | | Allopurinol  induced SJS/TEN  (n=6) | | Control *vs.* DRESS | Control *vs.* SJS/TEN |
| --- | --- | --- | --- | --- | --- | --- | --- | --- | --- | --- |
| HLA | N | % | N | % | N | % | N | % | *P* value | *P* value |
| A*02:01 | 7 | 13.5 | 5 | 18.5 | 4 | 19.0 | 1 | 16.7 | 0.719 | 1.000 |
| A*02:06 | 6 | 11.5 | 2 | 7.4 | 0 | 0.0 | 2 | 33.3 | 0.173 | 0.189 |
| A*02:07 | 4 | 7.7 | 2 | 7.4 | 1 | 4.8 | 1 | 16.7 | 1.000 | 0.433 |
| A*03:01 | 1 | 1.9 | 0 | 0.0 | 0 | 0.0 | 0 | 0 | 1.000 | 1.000 |
| A*11:01 | 13 | 25.0 | 2 | 7.4 | 1 | 4.8 | 1 | 16.7 | 0.054 | 1.000 |
| A*24:02 | 9 | 17.3 | 16 | 59.3 | 15 | 71.4 | 1 | 16.7 | <0.001 | 1.000 |
| A*24:08 | 1 | 1.9 | 0 | 0.0 | 0 | 0.0 | 0 | 0 | 1.000 | 1.000 |
| A*26:01 | 1 | 1.9 | 0 | 0.0 | 0 | 0.0 | 0 | 0 | 1.000 | 1.000 |
| A*26:03 | 2 | 3.8 | 0 | 0.0 | 0 | 0.0 | 0 | 0 | 1.000 | 1.000 |
| A*29:01 | 1 | 1.9 | 0 | 0.0 | 0 | 0.0 | 0 | 0 | 1.000 | 1.000 |
| A*30:01 | 0 | 0.0 | 1 | 3.7 | 1 | 4.8 | 0 | 0 | 0.103 | 1.000 |
| A*30:04 | 1 | 1.9 | 1 | 3.7 | 1 | 4.8 | 0 | 0 | 0.198 | 1.000 |
| A*31:01 | 2 | 3.8 | 1 | 3.7 | 1 | 4.8 | 0 | 0 | 0.284 | 1.000 |
| A*33:03 | 45 | 86.5 | 22 | 81.5 | 17 | 81.0 | 5 | 83.3 | 0.719 | 1.000 |
| B*07:02 | 0 | 0.0 | 2 | 7.4 | 2 | 9.5 | 0 | 0 | 0.080 | 1.000 |
| B*07:05 | 1 | 1.9 | 0 | 0.0 | 0 | 0.0 | 0 | 0 | 1.000 | 1.000 |
| B*13:02 | 1 | 1.9 | 1 | 3.7 | 1 | 4.8 | 0 | 0 | 0.496 | 1.000 |
| B*14:01 | 1 | 1.9 | 0 | 0.0 | 0 | 0.0 | 0 | 0 | 1.000 | 1.000 |
| B*15:01 | 9 | 17.3 | 4 | 14.8 | 2 | 9.5 | 2 | 33.3 | 0.494 | 0.318 |
| B*15:18 | 1 | 1.9 | 0 | 0.0 | 0 | 0.0 | 0 | 0 | 1.000 | 1.000 |
| B*35:01 | 6 | 11.5 | 1 | 3.7 | 1 | 4.8 | 0 | 0 | 0.665 | 1.000 |
| B*39:01 | 2 | 3.8 | 1 | 3.7 | 1 | 4.8 | 0 | 0 | 1.000 | 1.000 |
| B*40:01 | 3 | 5.8 | 4 | 14.8 | 2 | 9.5 | 2 | 33.3 | 0.622 | 0.078 |
| B*40:02 | 2 | 3.8 | 3 | 11.1 | 2 | 9.5 | 1 | 16.7 | 0.574 | 0.284 |
| B*40:06 | 0 | 0.0 | 2 | 7.4 | 2 | 9.5 | 0 | 0 | 0.080 | 1.000 |
| B*44:03 | 6 | 11.5 | 1 | 3.7 | 1 | 4.8 | 0 | 0 | 0.665 | 1.000 |
| B*46:01 | 5 | 9.6 | 2 | 7.4 | 1 | 4.8 | 1 | 16.7 | 0.666 | 0.497 |
| B*48:01 | 1 | 1.9 | 1 | 3.7 | 1 | 4.8 | 0 | 0 | 0.496 | 1.000 |
| B*51:01 | 3 | 5.8 | 2 | 7.4 | 2 | 9.5 | 0 | 0 | 0.622 | 1.000 |
| B*51:02 | 1 | 1.9 | 0 | 0.0 | 0 | 0.0 | 0 | 0 | 1.000 | 1.000 |
| B*52:01 | 1 | 1.9 | 0 | 0.0 | 0 | 0.0 | 0 | 0 | 1.000 | 1.000 |
| B*54:01 | 5 | 9.6 | 0 | 0.0 | 0 | 0.0 | 0 | 0 | 0.312 | 1.000 |
| B*55:02 | 1 | 1.9 | 0 | 0.0 | 0 | 0.0 | 0 | 0 | 1.000 | 1.000 |
| B*58:01 | 52 | 100.0 | 27 | 100.0 | 21 | 100.0 | 6 | 100 | 1.000 | 1.000 |
| B*59:01 | 0 | 0.0 | 2 | 7.4 | 2 | 9.5 | 0 | 0 | 0.080 | 1.000 |
| C*01:02 | 10 | 19.2 | 5 | 18.5 | 4 | 19.0 | 1 | 16.7 | 1.000 | 1.000 |
| C*01:03 | 1 | 1.9 | 0 | 0.0 | 0 | 0.0 | 0 | 0 | 1.000 | 1.000 |
| C*03:02 | 52 | 100.0 | 26 | 96.3 | 20 | 95.2 | 6 | 100 | 0.288 | 1.000 |
| C*03:03 | 8 | 15.4 | 4 | 14.8 | 4 | 19.0 | 0 | 0 | 0.734 | 0.581 |
| C*03:04 | 4 | 7.7 | 3 | 11.1 | 2 | 9.5 | 1 | 16.7 | 1.000 | 0.433 |
| C*04:01 | 7 | 13.5 | 4 | 14.8 | 1 | 4.8 | 3 | 50 | 0.425 | 0.057 |
| C*06:02 | 1 | 1.9 | 1 | 3.7 | 1 | 4.8 | 0 | 0 | 0.496 | 1.000 |
| C*07:01 | 2 | 3.8 | 1 | 3.7 | 1 | 4.8 | 0 | 0 | 1.000 | 1.000 |
| C*07:02 | 3 | 5.8 | 3 | 11.1 | 3 | 14.3 | 0 | 0 | 0.345 | 1.000 |
| C*07:04 | 1 | 1.9 | 0 | 0.0 | 0 | 0.0 | 0 | 0 | 1.000 | 1.000 |
| C*08:01 | 0 | 0.0 | 1 | 3.7 | 1 | 4.8 | 0 | 0 | 0.103 | 1.000 |
| C*08:02 | 1 | 1.9 | 0 | 0.0 | 0 | 0.0 | 0 | 0 | 1.000 | 1.000 |
| C*08:03 | 0 | 0.0 | 1 | 3.7 | 1 | 4.8 | 0 | 0 | 0.103 | 1.000 |
| C*12:02 | 1 | 1.9 | 0 | 0.0 | 0 | 0.0 | 0 | 0 | 1.000 | 1.000 |
| C*14:02 | 1 | 1.9 | 1 | 3.7 | 1 | 4.8 | 0 | 0 | 0.496 | 1.000 |
| C*14:03 | 4 | 7.7 | 1 | 3.7 | 1 | 4.8 | 0 | 0 | 1.000 | 1.000 |
| C*15:02 | 4 | 7.7 | 2 | 7.4 | 1 | 4.8 | 1 | 16.7 | 1.000 | 0.433 |
| C*15:05 | 1 | 1.9 | 0 | 0.0 | 0 | 0.0 | 0 | 0 | 1.000 | 1.000 |
| DRB1*01:01 | 1 | 1.9 | 3 | 11.1 | 2 | 9.5 | 1 | 16.7 | 0.197 | 0.198 |
| DRB1*03:01 | 18 | 34.6 | 3 | 11.1 | 2 | 9.5 | 1 | 16.7 | 0.042 | 0.653 |
| DRB1*04:01 | 0 | 0.0 | 1 | 3.7 | 0 | 0.0 | 1 | 16.7 | 1.000 | 0.103 |
| DRB1*04:03 | 2 | 3.8 | 3 | 11.1 | 3 | 14.3 | 0 | 0 | 0.140 | 1.000 |
| DRB1*04:05 | 6 | 11.5 | 3 | 11.1 | 2 | 9.5 | 1 | 16.7 | 1.000 | 0.555 |
| DRB1*04:06 | 3 | 5.8 | 2 | 7.4 | 0 | 0.0 | 2 | 33.3 | 0.552 | 0.078 |
| DRB1*04:10 | 1 | 1.9 | 0 | 0.0 | 0 | 0.0 | 0 | 0 | 1.000 | 1.000 |
| DRB1*07:01 | 2 | 3.8 | 0 | 0.0 | 0 | 0.0 | 0 | 0 | 1.000 | 1.000 |
| DRB1*08:02 | 1 | 1.9 | 1 | 3.7 | 1 | 4.8 | 0 | 0 | 0.496 | 1.000 |
| DRB1*08:03 | 9 | 17.3 | 3 | 11.1 | 2 | 9.5 | 1 | 16.7 | 0.494 | 1.000 |
| DRB1*09:01 | 5 | 9.6 | 7 | 25.9 | 5 | 23.8 | 2 | 33.3 | 0.139 | 0.149 |
| DRB1*10:01 | 2 | 3.8 | 0 | 0.0 | 0 | 0.0 | 0 | 0 | 1.000 | 1.000 |
| DRB1*11:01 | 3 | 5.8 | 1 | 3.7 | 1 | 4.8 | 0 | 0 | 1.000 | 1.000 |
| DRB1*12:01 | 4 | 7.7 | 0 | 0.0 | 0 | 0.0 | 0 | 0 | 0.318 | 1.000 |
| DRB1*12:02 | 3 | 5.8 | 1 | 3.7 | 0 | 0.0 | 1 | 16.7 | 0.552 | 0.362 |
| DRB1*13:02 | 28 | 53.8 | 18 | 66.7 | 16 | 76.2 | 2 | 33.3 | 0.113 | 0.416 |
| DRB1*14:01 | 2 | 3.8 | 0 | 0.0 | 0 | 0.0 | 0 | 0 | 1.000 | 1.000 |
| DRB1*14:05 | 2 | 3.8 | 1 | 3.7 | 1 | 4.8 | 0 | 0 | 1.000 | 1.000 |
| DRB1*15:01 | 5 | 9.6 | 4 | 14.8 | 4 | 19.0 | 0 | 0 | 0.269 | 1.000 |
| DRB1*15:02 | 1 | 1.9 | 0 | 0.0 | 0 | 0.0 | 0 | 0 | 1.000 | 1.000 |
| DRB1*16:02 | 3 | 5.8 | 0 | 0.0 | 0 | 0.0 | 0 | 0 | 0.552 | 1.000 |

Abbreviation

HLA, Human leukocyte antigen; DRESS, Drug reaction with eosinophilia and systemic symptoms; SJS, Stevens-Johnson syndrome; TEN, Toxic epidermal necrolysis

Footnotes

Allopurinol tolerant controls had no adverse reactions including cutaneous hypersensitivity during allopurinol taking for more than 90 days.

Table S3. Results of binary logistic regression to evaluate risk factors in considering clinical differences between B*58:01(+) allopurinol-induced DRESS and tolerant controls

|  | DRESS *vs.* Control | | | |
| --- | --- | --- | --- | --- |
|  | Univariate analysis | | Multivariate analysis^a^ | |
| Variables | OR (95% CI) | *P* value | OR (95% CI) | *P* value |
| Age ≥60 years | 2.0 (0.6-6.2) | 0.229 |  |  |
| Female | 4.6 (1.5-13.7) | 0.007 | 56.2 (3.1-1024.2) | 0.007 |
| BMI ≥25 kg/m^2^ | 2.0 (0.7-6.1) | 0.224 |  |  |
| Baseline eGFR <60 ml/min/1.73m^2^ | 0.1 (0.0-0.4) | 0.001 | 0.0 (0.0-0.2) | 0.002 |
| Dose of allopurinol ≥200 mg/day | 5.7 (1.2-26.9) | 0.028 | 319.8 (6.3-16260.4) | 0.004 |
| Cardiovascular disease | 2.3 (0.8-6.6) | 0.118 |  |  |
| HLA-A*24:02 (+) | 11.9 (3.6-39.2) | 0.001 | 55.0 (4.3-702.0) | 0.002 |
| HLA-A*24:02 (+) and DRB1*13:02 (+) | 66.0 (6.1-716.2) | 0.001 | NA |  |

Abbreviation

DRESS, Drug reaction with eosinophilia and systemic symptoms; OR, Odds ratio; CI, confidence interval; BMI, Body mass index; eGFR, estimated Glomerular fractional rate using CKD-EPI; HLA, Human leukocyte antigen; NA, not assessed

Footnotes

^a^Multivariate: gender, eGFR, dose of allopurinol and A*24:02; Presence of both A*24:02 and DRB1*13:02 was excluded in multivariate logistic regression analysis because the number was too small to be analyzed.
